# The Role of Teachers’ Interpersonal Behaviors in Learners’ Academic Achievements

**DOI:** 10.3389/fpsyg.2022.921832

**Published:** 2022-06-17

**Authors:** Qian Zhang

**Affiliations:** School of Humanities and Foreign Languages, Xi’an University of Posts and Telecommunications, Xi’an, China

**Keywords:** academic achievement, rapport, teacher’s interpersonal behaviors, teacher immediacy, teacher-learner interpersonal relationship, teacher stroke

## Abstract

In the context of English as a foreign language classroom, affections that form between teacher and students may affect the teaching/learning process. This review aimed to investigate the related studies on the effect of teacher-learner interpersonal relationships on learners’ educational performance in English as a Foreign Language (EFL) educational contexts. This review concluded that there is a significant constructive correlation between teacher-learner interpersonal relationships and learners’ academic achievement. Learners are required to have some sense of belonging to improve their educational performance. Moreover, other positive emotional factors such as grit, wellbeing, self-efficacy, academic engagement, motivation, and foreign language enjoyment can mediate the association between teacher-learner interpersonal relationships and learners’ academic success. The study concludes with some implications for English learners, English language teachers, and English language teacher trainers. The ideas can improve their awareness of teacher-student interpersonal relationships, including teacher stroke, rapport, and teacher immediacy and their role in improving learners’ foreign language learning.

## Introduction

Krashen (1981), in his theory of second language acquisition, introduced the Affective Filter hypothesis, the theory that concerns the importance of the affective factors as one of the main elements in second language learning. He argued that “the best methods are, therefore, those that supply “comprehensible input” in low anxiety situations, containing messages that students really want to hear” (p. 120). He maintained that the way the teacher and students interact with each other in the class can have an effect on students’ feelings about the teacher, class, and even language learning. The way teacher reacts in-class questioning can be one of the factors that change students’ emotions in the classroom and affects the interpersonal relationship between teachers and students as well. Based on Krashen (1981), the affective factors may have a key role in teacher-student interaction and eventually in language acquisition. Krashen (1981) believed that students with inspiration, confidence, and low levels of anxiety are anticipated to be more successful in learning a new language. According to Zembylas (2015), emotions are “a form of consciousness lived, experienced, articulated, and felt.” (p. 186). He asserted that emotions influence people’s behavior, their beliefs, and their relationships with others in the different contexts in which they live, study, and work.

Until recently, attention to emotions was not taken into account as a must in educational contexts. As Hargreaves (1998) argued, “The reason was the distance of the emotions from reasoning so affections did not play a role in the classroom” (p. 560). Tyng et al. (2017) indicated that emotion has a substantial influence on the cognitive processes in humans, including perception, attention, learning, memory, reasoning, and problem solving. Nowadays, a positive relationship in the classroom is considered an influential factor in language learning since it can have either a positive or negative effect on students’ achievement and willingness to class work. It may also improve their knowledge and social skills (Larson, 2011). Furthermore, Nugent (2009) asserted that when the emotional state of wellbeing is shaped in the educational context, the motivation of learners during the learning process will be simple. Positive teacher-learner interpersonal relationships, thus, can be expected to have an influence on learners’ educational performance. Then, it is necessary for the teachers to make students feel comfortable and confident with their teacher. That is, teachers need to be aware of the impact of the emotional dimension and affection domain in their EFL classroom (Wu, 2019).

Generally, instructors have a longstanding influence on their students once they specify the way of learners’ understanding and their interaction with society (Han and Xu, 2020). Any investigation of learners’ academic skills should consider the teacher-learner rapport, stroke, personality, ethical standards, instructional designs, and positive viewpoints are vital (Gao et al., 2019). The closeness between learners and educators will boost the efficacy of the L2 context and support them to reach improved results (Pham, 2019). The achievement or incompetence in improving language learning depends on some particular vital variables, including social and cultural environment, nature, and instructional method, and contextual features (Torres et al., 2020). Numerous investigations were done on teacher-learner interpersonal relationships along with notable efforts to understand if this affects the levels of learners. This review tries to investigate the studies on the effect of teachers’ interpersonal behaviors and learners’ academic performance. The significance of this study is to raise educators’ awareness of their emotional behaviors and their impacts on students’ educational attainment.

## Literature Review

### The Notion of Teacher-Learner Interpersonal Relationship

Brinkworth et al. (2018) defined the teacher-student interpersonal relationship as teachers’ and students’ combined and progressing insights over each other, which affect the interaction longitudinally. They mentioned that these insights are kept in memory and contribute to the upcoming interactions. They also asserted that when learners perceive the support and the reliability of instructors, they tend to interact with their educators and consider their teacher as someone who supports them and provides them with some hints to develop their education. Davis (2003) pointed out that effective instruction comprises high-quality interaction between educators and learners and also among peers. He asserted that appropriate outcomes appear when collaboration between educators and learners occurs. Therefore, teachers play a significant role in educational contexts. Alzeebaree and Zebari (2021), in their studies on the causes of effective teaching, mentioned that teachers should be supportive, provide promising educational contexts and inspire learners to attempt in language learning. Furthermore, there is an established belief that the relations that learners expand in educational contexts between teachers, peers, and principals, lead to individual, educational, and social development (Pakarinen et al., 2018). In effect, when learners start their educational tasks, the relations they develop with their instructors become gradually significant for their achievement in educational contexts (Heatly and Votruba-Drzal, 2017).

Some approaches have been employed to explicate the relationship between learners and teachers. For instance, Bergin and Bergin (2009) attachment-based approach to teacher-learner interpersonal relationships is on the premise that educators, who provide sincere, secure, and helpful interaction with their learners, can act as central non-parental attachment persons. Verschueren and Koomen (2012) explicated that when learners feel safe, they can delve into the educational contexts and involve in educational tasks and activities without any stress. They also mentioned that intimacy is theorized as the extent of openness, warmth, and friendship in the communication between teachers and students. Creasey et al. (2009) categorized the behaviors of teacher-learner interpersonal relationships into four groups: connectedness, nervousness, independency/dependency, and peaceful/conflicting. They mentioned that connectedness is related to the intimate behaviors between learners and educators. They also defined nervousness as the level of learners’ discomfort with their educators. Moreover, they maintained that the dependent/independent relationship is the learners’ emotion of independency or dependency with their instructors. They asserted that peacefulness develops in strong relationships, whereas conflict occurs in undesirable relationships. In general, according to Pianta et al. (1995), “a close relationship is characterized by warmth, open communication, and support, while a conflicted relationship is characterized by anger, and hostility” (p. 28).

Teacher-learner interpersonal relationships and the significance of this relationship among learners have been widely studied (Violanti et al., 2018). The quality of teacher-learner interpersonal relationships can affect some positive psychological constructs, including academic engagement, foreign language enjoyment, resilience, grit, self-efficacy, and wellbeing among learners in order to improve learners’ healthy behavioral functioning (Derakhshan, 2021). Concerning learners’ academic engagement, Dennie et al. (2019) found that teacher-learner interpersonal relationships significantly affected learners’ academic engagement in educational contexts. They argued that teacher-learner interpersonal relationship positively affects learners’ emotional requirements, which influence academic engagement. Derakhshan et al. (2022) investigated the effect of teacher care and teacher-student rapport on Iranian and Polish L2 learners’ academic engagement in L2 context. Their study demonstrated that learners’ viewpoints about cultural and instructional contexts influenced their engagement. Their study empirically reinforced the belief that engagement is the by-product of the dynamic interaction between the individual factors and the instructional context. Jia et al. (2020), in their study, revealed that learners’ academic engagement is significantly correlated with educators’ supportive interaction. They argued that educators’ use of passionate, warm interaction, and reverence would improve student satisfaction which contributes learners to being more involved in educational contexts. Engels et al. (2021), in their study, revealed that closeness and conflict, as the constructs of teacher-learner interpersonal relationship, respectively, positively and negatively affected learners’ academic engagement. They mentioned that learners’ feeling about the support of teachers can inspire them to engage socially, academically, and emotionally in educational contexts.

Regarding learners’ foreign language enjoyment, Song (2021) found that teacher stroke as a positive teacher communication behavior, has a significant role in developing positive psychological constructs, like learner foreign language enjoyment. The results of Mainhard et al. (2018) revealed that teacher agency (interactive dominance or influence) and empathy (warmth or intimacy) are important for developing learners’ positive emotions like enjoyment. Goetz et al. (2021) mentioned that a teacher-learner interpersonal relationship with higher quality is correlated with enjoyment, while foreign language anxiety is negatively correlated with less efficient teacher-learner interpersonal relationships. They argued that learners’ decision about high relational intimacy to the educator improves their management of situations which can adjust their enjoyment.

Regarding grit, Yuan (2022), in his study, revealed that teachers’ communication behavior, such as teacher stroke and teacher-student rapport significantly affect learners’ grit. He argued that teacher-student rapport and teacher stroke is related to students’ grit. In other words, a positive teacher-learner interpersonal relationship can enhance students’ eagerness, the achievement of central interpersonal skills, and commitment, together with a decrease in their nervousness level that results in more grit among learners. Ma et al. (2020) also mentioned that teachers’ support as a communication behavior significantly affects learners’ grit. They argued that teacher-student positive relationship fosters learners’ social competence. Lan and Moscardino (2019) indicated that a teacher-student positive relationship and grit significantly affected learners’ wellbeing. They also asserted that “the buffering effect of grit on the association between teacher-student interpersonal relationship quality and student wellbeing is independent of parental migration” (p. 131).

The concept of teacher-student interpersonal relationships has been overlooked among the models of learner motivation. However, little research has been done on the relationship between teacher-learner relations and learner motivation. Pishghadam and Khajavy (2014) stated that “when educators pay attention to their learners and ask them to take part in classroom activities, learners can gain a higher level of motivation and better performances” (p. 6). Rajabnejad et al. (2017) asserted that teachers’ positive behaviors are significantly correlated with L2 learners’ motivation and inclination to join classes. Meng (2021), in his study, found out that teacher-learner rapport leads to learner engagement and learner motivation. Using relational goal theory, he argued that the significance of learner-teacher rapport encourages educators to use the modern methodology and reconsider their opinions to build positive rapport with learners to enhance motivation. It must be stressed that educators who have a positive rapport with their students engender educational context to develop learning and students’ emotional positive constructs. Henry and Thorsen (2018) also found out that teacher immediacy significantly affects learner engagement and state motivation. They argued that in developing interactions, moments of interaction can create instant, conscious responses that lead to increased motivational energy. Houser and Hosek (2018) used rhetorical and relational goal theory in order to justify their findings about the relationship between motivation and teacher-learner interpersonal relationships. They argued that “based on rhetorical and relational goal theory, when instructors utilize efficient interpersonal communication cues to meet learners’ relational and rhetorical wants, learners are more likely to experience a wide range of desirable outcomes including learning, interest, engagement, empowerment, motivation, and achievement” (p. 21).

Regarding learner self-efficacy, Li and Yang (2021) have found out that teacher-learner interpersonal relationships can improve learner-self-efficacy in Chinese contexts. They argued that teacher-learner positive interpersonal relationships can raise learners’ inclination to the educational contexts that foster their self-efficacy. Hajovsky et al. (2020) also found a reciprocal relationship between learner self-efficacy and teacher-learner rapport. They argued that educators with higher self-confidence in their aptitudes to instruct, evaluate, and accomplish educational tasks tend to be more involved in educational activities that result in supportive and secure relationships with learners that improve learner self-efficacy. Velez and Cano (2012) asserted that teachers’ immediacy, as a positive teacher behavior with learners, can significantly predict learner self-efficacy. They used Bandura (1997) theory about self-efficacy in order to justify their results. According to this theory, there are four reasons, including mastery experiences, vicarious experiences, verbal persuasion, and physiological and affective states, which improve learner self-efficacy. They mentioned that the non-verbal relationship between teacher and learners is one of the most important factors which affects learner self-efficacy.

Last but not least, an important affective construct that is significant in learners’ academic achievement is wellbeing. Holfve-Sabel (2014) investigated the predictability of teacher-learner rapport in learners’ wellbeing. Their findings indicated that a positive relationship between teacher and learners can significantly predict learners’ wellbeing in instructive contexts. Li (2022) found out that teacher-learner rapport can significantly increase learners’ wellbeing. He employed Mottet et al. (2006) rhetorical-relational goal theory in order to elucidate the relationship between wellbeing and teacher-student rapport. He argued that educators can arrange for pleasurable educational contexts via numerous relational and rhetorical communication behaviors such as rapport. In pleasant educational contexts, learners feel positive emotional constructs, such as enjoyment, pride, and hope which are associated with learner wellbeing. Luo et al. (2020) also indicated that a teacher-learner positive interpersonal relationship offers a non-pressured context in which learners’ wellbeing can be intensely developed. They argued that teacher-learner positive interpersonal relationship makes learners alleviate their pressure, nervousness, and anxiety that are harmful to their psychological wellbeing. Poulou (2020), in his study, revealed that teacher-learner rapport was the strongest predictor of learner wellbeing. They also found that teacher-learner rapport mediates the correlation between educators’ emotional need satisfaction and learners’ emotional and social problems.

### The Effect of Teachers’ Interpersonal Behaviors on Learners’ Academic Achievements

According to Bates et al. (2013), learners’ academic success is defined as their academic capabilities and is regularly determined using learners’ standardized test scores. Academic achievement is widely studied because of its theoretical and applied implications. Academic achievement is related to high levels of learner success with publicly appropriate results, including work accomplishment and performance (Kanfer et al., 2010). Some studies have been done on the relationship between teacher-learner interpersonal relationships and academic achievement. Lowman (1995), in his effective instruction model, asserted that teacher-learner positive relationship “deals with an instructor’s awareness of these [classroom] interpersonal phenomena and with his or her skill as communicating with students in ways that increase motivation, enjoyment, and predictor learning” (p. 27). Roorda et al. (2019) found out that teacher-learner immediacy enhances learners’ academic success. They argued that learners’ success in educational contexts can be the result of teacher-learner interaction. Engels et al. (2019) also found that negative teacher teacher-learner interpersonal relationship, which results in conflict, can impede learners’ academic achievement. They theorized conflict as the extent of negative teacher-learner interactions. Tawana (2020) investigated the influence of teacher-learner rapport on learner’s learning at secondary educational contexts. He stated that teacher’ insights about the teacher-learner interpersonal relationship can influence the quality of teaching, which shape learners’ emotional engagement in educational contexts and their educational success. Chamizo-Nieto et al. (2021), in their study, revealed the mediating effect of teacher-learner relation in the correlation between emotional intelligence and academic achievement. They argued that the strong teacher-learner interpersonal relationship causes the intelligent learners to be successful in academic contexts.

Mensah and Koomson (2020) asserted that learners who identify their educators as more helpful have academic achievement. They argued that teachers should be involved in communication with their learners in order to foster language learners’ capabilities. They also mentioned that “whereas a positive teacher-student interpersonal relationship creates an enabling environment for academic engagement and achievement, the lack of it stifles the level of academic engagement and achievement to some extent. Students require some sense of belonging to enhance their academic work” (p. 107). The study of Ansari et al. (2020) revealed that educators with positive relationships with learners have learners with moderately academic success in educational contexts. They also found that teacher-learner negative interpersonal relationship leads to underachievement, and inconsistency in teacher-learner interpersonal relationship is not constantly correlated with learners’ academic performance. They argued that teacher immediacy enhances learner involvement, which has positive results for attention and doing educational tasks.

Derakhshan et al. (2019) recognized teacher stroke as one of the factors which inspire learners to be able to achieve in educational contexts. They asserted teacher stroke is related to the higher levels of learner commitment that accordingly result in the improvement of the interest among learners and higher levels of academic success. Baños et al. (2019) also mentioned that educators who have higher levels of stroke and positive relationships with learners can improve the obligation of the learners to be successful in a classroom environment. Bhatti et al. (2020) declared that academic success or failure is related to important features like the students’ social and cultural context, character, learning process, and teacher-learner interpersonal relationship. Mabunda and Mulovhedzi (2020) also mentioned that the intimacy between educators and students has central importance in the learners’ educational performance. They argued that “educators can also offer some activities to learners chosen as class representatives, and which allows them to feel a sense of belongingness and being valued in the learning context” (p. 20). This consequently helps the development of learning among learners. According to Hallinan (2008), “learning is a process that contains cognitive and social dimensions which need to be well-thought-out if academics success is to be exploited” (p. 271). Irungu et al. (2019) verified the efficient use of teacher non-verbal immediacy during instruction and education. They maintained that teacher immediacy boosts the quality of learning, which, in turn, results in higher educational achievement. Their study implicated that teachers should apply non-verbal immediacy as an instructional technique in order to develop learner success. Behjat et al. (2014) demonstrated that teachers’ facial expressions and eye contact in communicating with the learners are significant in developing learners’ education. They argued non-verbal immediacy like eye contact can activate the learning context by raising learners’ consciousness in educational contexts. This leads to engagement among learners, which develops learners’ working memory and academic achievement.

Lammers and Byrd (2019) verified the association between teacher-learner positive relationships and learners’ educational success. They argued that learners with higher intimacy levels are inclined to have higher scores. However, they mentioned that “these outcomes are not significantly better than those of students whose rapport with their instructors increased over time” (p. 42). Estepp and Roberts (2015) found that the quality of a professor-student rapport can predict student motivation and engagement. The participants in their study agreed they had a good rapport with their professors because those professors since they utilized non-verbal and verbal immediacy behaviors. They found that the correlation between immediacy/rapport and motivation was important for a positive student outcome. Demir et al. (2019) also indicated that teacher-learner rapport and autonomy support significantly predict learner achievement. They argued that, teacher-learner rapport leads to motivation, which, in turn, increases learner achievement. They also mentioned that teacher-learner rapport enhances learners’ insights over the teacher efficiency and their achievement. Hussain et al. (2021) examined the correlation between learners’ perceived teacher immediacy and their motivation. They asserted that positive contact and teacher intimacy leads to higher motivation and achievement among learners. They mentioned that motivated learners, who perceive teacher immediacy, are prone to be diligent in their attempts, and learn better in educational contexts.

Sutiyatno (2018), in his study, concluded that verbal and non-verbal immediacy are important in instruction and education. He argued that educators are required to foster and improve effective communication to positively convey educational materials to the learner. Bunglowala and Bunglowala (2015, p. 371) indicated that “teachers are required to learn to use non-verbal communication to improve classroom teaching.” Furthermore, Salimi (2014) found that the learners’ positive viewpoints about English vocabulary and reading comprehension is significantly correlated with teachers’ non-verbal immediacy. According to Mortazavi (2013):

“expression of words vividly and eloquently by the teacher causes the students to listen with dignity and willingness, sum up the facts, and think to solve the problem and supply the teacher’s comment without any tensions or boredom. But using biased phrases often raises a sense of stubbornness and humiliation in the trainee, puts them out the cycle of learning and deep understanding of scientific content completely, and leaves irreparable psychological effects on the students” (p. 21).

Some investigations could not verify a significant association between teacher-learner interpersonal relationships and educational success achievement. Mohamed et al. (2018) used closeness, conflict, and dependency as three components in the Student-Teacher Relationship Scale inventory. Their study demonstrated a moderate, but insignificant relationship between student-teacher rapport and learners’ academic achievement. A study of 182 students of a high performing and a low performing high school by Nugent (2009) showed a weak positive correlation between teacher-student rapport and overall GPA. However, the study found a significant positive relationship between teacher-student rapport and motivation. Similarly, a recent study by Mabin (2016) using a sample of more than 3,000 high school students did not show significant findings in the correlation between student-teacher rapport (e.g., a caring teacher) and academic achievement. A few studies on older students also did not indicate a significant correlation between student-teacher rapport and academic achievement. Bin Abdulrahman (2007), for example, found no correlation between medical students’ academic grades and their views on their relationship with their teachers, while Nyadanu et al. (2015), using a sample of nursing students, only found a weak correlation between student-teacher rapport and academic performance.

Zheng (2021), in another study, argued that clarity and immediacy, as two behavioral indicators of instructors, are two associated components that significantly affect teachers’ cognitive ability and emotions, respectively. He mentioned that teacher clarity activates learners’ cognitive capability, and teacher immediacy provokes learners’ affections. He maintained that teacher immediacy and clarity support learners’ academic success. He also used Paas et al. (2003) additivity hypothesis, which indicated that two elements, in combination, shape an educational environment for the students.

## Final Remarks

This review investigated the effect of teachers’ positive behaviors like the positive interpersonal relationship with learners on learners’ academic achievement. It concluded that learners may experience the feeling of being more contented and inspired to learn when educators have a positive rapport with them, and encourage them to engage more in educational contexts. Overall, to build an interpersonal relationship, a combination of empathy, respect, and patience is necessary. This review indicated that teacher rapport leards to the increase in motivation and enjoyment, which in turn build up language learning. It also showed the studies on the positive relationship between teacher-learner positive interaction and learner achievement. Moreover, it also pinpoints the influential role of teachers’ viewpoints about the teacher-learner interaction in the their instructional quality. Moreover, this review showed the positive role of teacher immediacy in learners’ academic engagement in educational contexts. A new concept like teacher stroke was also introduced in this review, which is an important factor in learner achievement. This review may help to support students’ inspiration and enhance their learning procedure. This review improves the informative knowledge of investigators who are interested in learners’ and teachers’ affections, and the causes of learners’ achievement. Regarding the related literature on the positive role of teachers’ interpersonal behaviors, including teacher immediacy, stroke, and rapport, in learners’ academic performance, it is worth noting that instructors should be assisted to handle their emotional states in educational environments. Learners are able to regulate their feelings to strengthen their academic performance, and this issue can stimulate instructors to take into account learners’ positive emotions in classroom contexts. So dividing the class into small groups or pair work might help if it is controlled carefully. Teacher stroke, like the feedback that students get from a teacher, will greatly influence English learners. Some teachers like to correct all the students’ language mistakes; it can be a reason to discourage students’ learning and interest that can harm effective factors. The growth of teachers’ interpersonal behaviors and learners’ academic performance, can be considered one of the pedagogical implications of this review. Learners can employ opportunities that instructors provide to express their thoughts and emotions, which can decrease their stress in educational contexts and inspire them to be responsible for their learning.

This study can also encourage teachers, teacher educators, and policymakers to consider EFL learners’ behaviors and their foreign language achievement. Also, understanding learners’ personality behaviors may inspire instructors to bring enthusiasm to EFL learning contexts. Therefore, it is necessary for L2 teachers to realize learners’ different types of motivation, and obviate their problems to improve learners’ points of view, motivation, enjoyment, and engagement in order to learn better in educational contexts. Teachers are required to reconstruct and modify the instructional materials. They can choose interactive ones to foster learners’ foreign language learning to attain better results. Incorporating technology into classroom contexts can be a pleasant way to increase learner foreign language learning. Using games and role play techniques in the classroom can also arouse teacher-learner rapport and improve language learning. This can diminish learners’ cognitive load and despair, and stimulate their enthusiasm and concentration on language learning contexts. A teacher can incorporate positive emotions in their methodology to increase learners’ foreign language learning. Thus, they can cater to warming-up tasks for learning contexts and brainstorm with learners to increase improve foreign language learning. The challenging projects, lectures, conferences, and workshops may lead to teacher burnout in academic contexts and put additional strain on learners. Teachers can manage the time of classrooms regularly to provide time for learners to communicate and outperform in educational contexts. Providing a competitive educational context through quizzes boosts learner engagement and learning.

Policymakers, consulters, and teacher educators should take into account the centrality of the student-teacher relationship in their professional activities regarding teacher education programs. Positive and constructive teacher-student interpersonal relationships should become an essential part of a language education program. Policymakers can develop programs that help teacher have a positive relationship with language learners and amplify their academic performance. They can hold academic workshops to help teachers control emotional behaviors. They can provide interesting facilities, technology and positive learning contexts for enticing learners and increasing the motivation of teachers to interact with learners. The importance of teacher-learner rapport may make consultants expand their agendas to diagnose teachers’ and learners’ lack of interest in the relationship, and the obstacles to learners face during language learning. They can also offer some suggestions to increase the efficiency of teacher-learner interpersonal relationships and learning performance.

Besides, teacher educators can exploit the related studies by considering the instructors’ positive relationship with the learners. They can hold workshops and provide some strategies to improve teacher immediacy. They can also emphasize modeling teacher-learner rapport, taking action and improving listening, and trying not to interrupt learners while they are speaking. They can give some instructions to use gestures in communication with learners. Moreover, some recommendations, such as using non-monotonous speech, smiling during the speech, looking at the whole class during talking, and having a relaxed posture, can be presented in the workshops. Teacher educators can assess and validate the effectiveness of instructors’ rapport on learners’ achievement in EFL contexts. They should improve self-confidence and capability among instructors to arouse learners’ interests and involvement in the learning process.

The present review introduces several research topics that could be taken up in future research. Future studies can be related to the effect of teachers’ cognition on teacher-student interpersonal relationships and to various approaches to building a constructive teacher-student relationship that would influence learners’ learning performance. Future studies may consist of investigating the influence of other teacher variables such as extroversion and introversion on learners’ positive behaviors such as enjoyment, grit, pride and engagement. Teacher’ negative emotion such as boredom, burnout, apprehension, and exhaustion and their role in learner foreign language enjoyment should be examined. Other studies can be done to investigate the effect of teacher stroke on learners’ academic engagement and enjoyment.

Investigation of teachers’ additional strategies for enhancing learners’ learning through teacher-student interaction can be studied in the future. Additionally, a future study can be done to systematically explore learners’ beliefs about the teacher-student relationship. Longitudinal studies are required to shed light on the effect of interpersonal emotions on language learning and learners’ positive emotions. Future studies can investigate the effect of the teacher-student positive relationship on learners’ working and long-term memory. Moreover, the effects of positive and negative teacher-learner interpersonal relationships on the improvement of language skills ought to be considered in detail. Further studies are needed to determine the teacher-learner interpersonal relationship in traditional and digital contexts to illuminate how these contexts may affect learners’ academic achievement. Further research can be done to investigate the gender effect and teacher proficiency level on teacher-learner interpersonal relationships in language learning contexts. Finally, future studies should determine the relationship between EFL teachers’ immediacy and emotional intelligence in foreign language contexts.

## Author Contributions

The author confirms being the sole contributor of this work and has approved it for publication.

## Conflict of Interest

The author declares that the research was conducted in the absence of any commercial or financial relationships that could be construed as a potential conflict of interest.

## Publisher’s Note

All claims expressed in this article are solely those of the authors and do not necessarily represent those of their affiliated organizations, or those of the publisher, the editors and the reviewers. Any product that may be evaluated in this article, or claim that may be made by its manufacturer, is not guaranteed or endorsed by the publisher.
